# New Products

**DOI:** 10.1155/S146392460000002X

**Published:** 2000


Journal of Automated Methods & Management in Chemistry, Vol. 22, No. 1 (January–February 2000) pp. 29–32

### New products

### C.I. expands sorption testing service

C.I. Electronics has expanded its sorption test facility, providing the chemical industry with a fast, cost-effective route to high-quality information on the water sorption behaviour of a wide range of materials. The new, purpose-built laboratory provides a controlled environment for sorption testing which enhances C.I.'s development facilities as well as increasing threefold the capacity of its testing service. It affords ideal conditions for measurements of the highest accuracy and consistency, while C.I.'s CISorp technology for automated analysis offers substantial productivity gains to companies currently employing manual measurement techniques.

Existing users of C.I.'s testing service benefit from the vast experience of a world leader in microbalance measuring technology. Ensuring that clients derive maximum value from its expertise in measurement of adsorption/desorption isotherms and kinetic testing, C.I. offers the services of a dedicated scientific consultancy team which will assist with optimization of the test regime. Following the investigation, the company supplies the raw data plus a detailed report containing experimental results and plots, together with an informed interpretation of results.

*For further information contact Elaine Roberts, C.I. Electronics Limited, Brunel Road, Churchfields, Salisbury SP2 7PX, UK; Tel: +44 (0)1722 336938; Fax: +44 (0)1722 323222; e-mail: admin@cielec.com; website: http://www.cielec.com*

### Faster, easier qualitative determination of fragrance compounds in hops from Shimadzu 'headspace technology'

The use of scientific techniques for the analysis of fragrance compounds in hops has long been part of the brewer's art. A method using the headspace technique for gas chromatography and mass spectrometric analysis has been introduced by Shimadzu—one of the world's leading manufacturers of analytical instruments—which is proving a powerful tool for qualifying fragrance substances.

Because of the easy sample handling associated with this technique, mistakes in sample preparation are avoided and, as analysis is performed with an autosampler, the method saves time compared to extraction. Quantification can also be performed using several calibration methods, for greater ease and user-friendliness.

Another benefit of the headspace method is the prevention of contamination of the injector and prolonged life of the column.

Using the headspace approach in a recent Shimadzu test, the effect of different conditioning temperatures was studied using two samples.

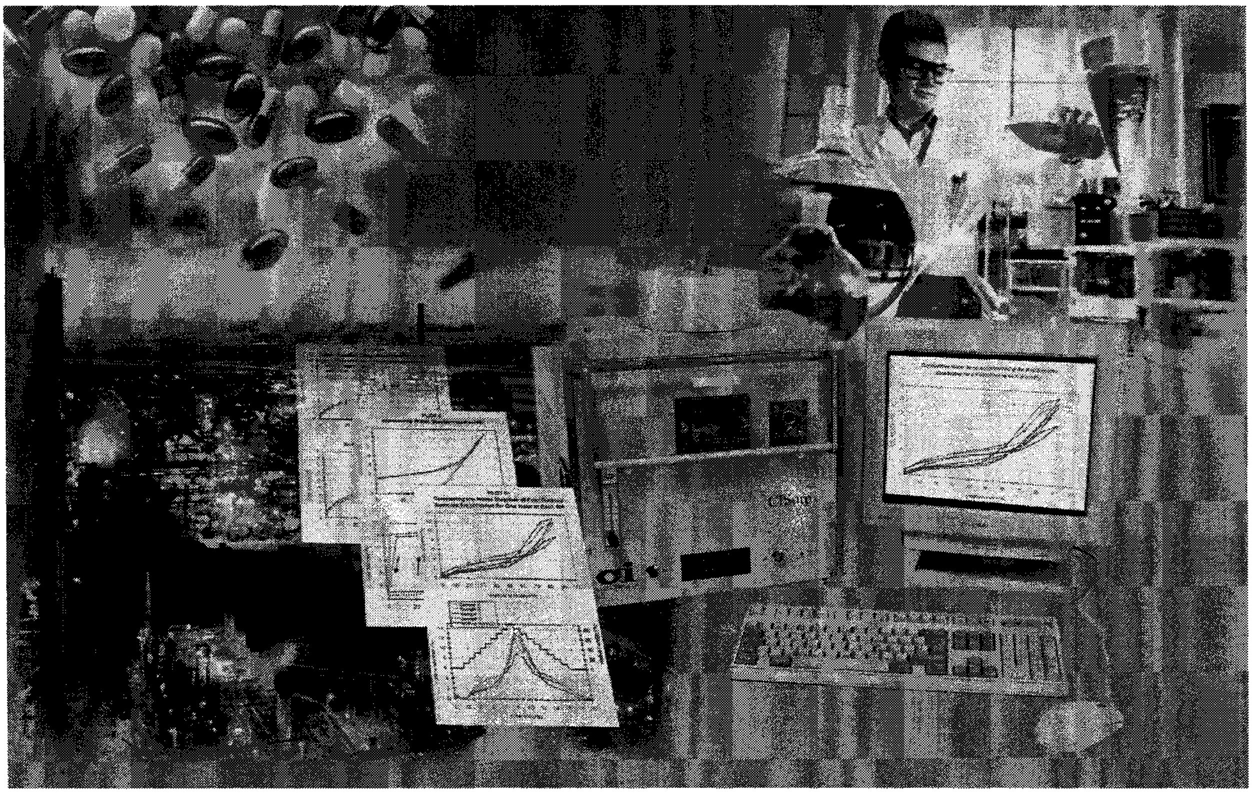

Under normal analysis protocols, 2 g of solid hop sample or granulate is added to a vial which is sealed gas-tight with a septum and cap. The sample is then conditioned until an equilibrium state is reached, which will depend on the conditioning temperature, the compounds to be analysed and the conditioning time, all of which need to be determined experimentally.

In this test, the samples were conditioned for 45 min at 80 and 120°C respectively, in order to compare results.

At the higher temperature, the peaks revealed by gas chromatography–mass spectrometry (GC–MS) analysis are much increased, and some compounds are much more easily detected. Because the fragrance substances characteristic for hops—delta-cadinene, farnesene and humulene—elute after 20 min, a higher conditioning temperature can therefore be seen to ensure maximum sensitivity.

Some fragrance substances show nearly the same fragmentation pattern, so it is useful to work with private libraries and retention indices, which will give useful information for spectra interpretation, as well as commercial libraries, e.g. NIST 75.000.

It is particularly advisable to use GC–MS techniques when a number of samples need to be compared, as this will give greater speed and accuracy. It is possible to subtract the spectra of two peaks in a special comparison window, e.g. to show quickly and easily whether the two subtracted compounds are identical or if there is coelution at the same retention time.

Qualitative analysis for single compounds can be performed very easily using multi-ion chromatograms. For alpha-humulene, e.g. typical mass traces m/z 93, m/z 80 and m/z 121 can be used to identify the substance easily, along with the typical ratio of the masses. This information is available in the libraries.

*For more information, please contact: Shimadzu UK, Mill Court, Featherstone Road, Wolverton Mill South, Milton Keynes MK12 5RE, UK: Tel: +44 (0)1908 552200; Fax: +44 (0)1908 552211; e-mail: sales@Shimadzu.co.uk or Ross Heaven, Strong Words, 32 Cranstoun Street, Northampton NN1 3BH, UK; Tel/Fax: +44 (0)1604 250221; e-mail: rossheaven@aol.com*

### Ensuring appealing taste and aroma in onion-based food products

Maintaining the aroma and taste of freshly cut onions is important for food products and preparations based on an onion content, and for greater consumer appeal of salad and cooking onions themselves.

This distinctive aroma derives from a high content of volatile compounds within the onion—scientifically known as (E)-(+)-S-(1-propenyl)-L-cystein sulphoxyd. The addition of intermediate products then enables multiple chemical processes to take place so that numerous end-products can be built from this base.

Shimadzu—one of the world's leading manufacturers of chromatography and mass spectrometry instruments—offers a procedure using its QP-5000 mass spectrometer, GC-17A gas chromatograph, HSS-4A headspace sampler and NIST 75.000 spectra library of flavours and fragrances, which enables the sulphur content of vegetables to be determined accurately and in a fully automated way to ensure perfect end results in food manufacture, blending and processing.

Using this technique, the material to be investigated is first cut up coarsely and 5 g added to a headspace vial. It is important not to cut up the material too finely, or substantial and uncontrollable losses of active substances would result from the reaction of residues with the acids of the plant tissue. Analysis should follow quickly from sample preparation so that results are reproducible in subsequent analyses.

Chromatographic conditions for analysis are a conditioning temperature of 80°C for 60 min with a detector temperature of 260°C and an injection temperature of 210°C at a volume of 0.8 ml. An extensive purge and trap procedure is not necessary as the technique ensures the measurement of all searched components with high sensitivity, with individual peaks assigned quickly and accurately using mass spectroscopy. Significant mass traces can also be underlayed beneath the total ion chromatogram to facilitate the search routine.

Private flavour and fragrance libraries as well as those commercially available can be integrated into the search, which is ideal for analysis of compounds in particular food manufacturing applications.

The Shimadzu technique is highly accurate and sensitive, allowing manufacturers consistency of aroma and flavour in food products. Better still, the process is fully automated so analysis time and costs are considerably reduced.

*For more information on this application and for details of other Shimadzu products for food manufacturing and processing, contact: Shimadzu UK, Mill court, Featherstone Road, Wolverton Mill South, Milton Keynes MK12 5RE, UK; Tel: +44 (0)1908 552200; Fax: +44 (0)1908 552211; e-mail: Sales@-Shimadzu.co.uk; Ross Heaven, Strong words, 32 Cranstoun Street, Northampton NN1 3BH, UK; Tel/Fax: +44 (0)1604 250221; e-mail: rossheaven@aol.com*

### Spectrum TimeBase software

Spectrum TimeBase is an exciting new time-resolved software package offering improved 3D graphics, fast calculations and new data viewing options. It can be used for data collection and display in GC-IR, TG-IR, kinetics, reaction monitoring, LC-IR and GPC-IR studies.

Spectrum TimeBase will control time-resolved data from a wide range of Perkin-Elmer IR instruments, including the Spectrum One, Spectrum GX, Spectrum 2000, System 2000, Spectrum RX, Spectrum BX, Spectrum 1000 and Paragon 1000 instruments.

*For further information, contact Carole-Anne Green, Perkin-Elmer Limited, Post Office Lane, Beaconsfield, Bucks HP9 1QA, UK; Tel: +44 (0)1494 676161 extn 3269; Fax: +44(0)1494 679331/3; e-mail: greenca@eur.perkin-elmer.com; website: http://www.perkin-elmer.com*

### Synthesis Monitoring System

The use of solid-phase synthesis in combinational chemistry is now established as an exciting new technique in the drug discovery process. It meets the chemist's requirement for rapid screening of reaction products whilst they are still bound to the solid phase resin. Perkin-Elmer's new Synthesis Monitoring System, based on FT-IR spectroscopy, allows rapid analysis from a few reaction beads. The expert software automatically compares spectra from 'before' and 'after' reaction stages, with the spectral information being correlated with functional group information. Reaction optimization can also be aided by estimating the percentage completion of the reaction.

This new monitoring system will be of particular interest to all scientists working in the fields of combinational chemistry and drug development.

*For further information, contact Carole-Anne Green, Perkin-Elmer Limited, Post Office Lane, Beaconsfield, Bucks HP9 1QA, UK; Tel: +44 (0)1494 676161 extn 3269; Fax: +44 (0)1494 679331/3; e-mail: greenca@eur.perkin-elmer.com, Website: http://www.perkin-elmer.com*

### Analytical instrument nitrogen generator will serve primary applications for LC/MS and solvent evaporation

An analytical nitrogen generator that produces purity levels of dry nitrogen of 99–99.9% has been designed by Whatman International for the specific purpose of supplying LC/MS instruments as well as for solvent evaporation (see photo on right).

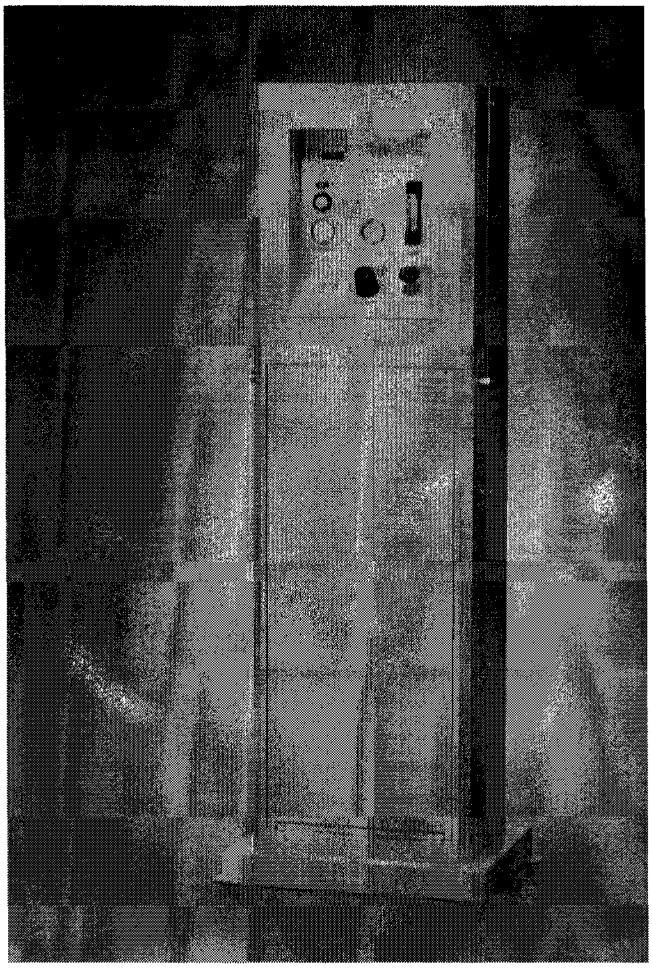

To operate the Type N2-2010 generator, it only requires to be connected by a $\frac{1}{2}''$ inlet connection to a supply of compressed air ranging from 4.2 to 10 bar to produce a maximum flow rate of nitrogen of 62 l min$^{-1}$. Payback on nitrogen systems can typically range from six months up to two years.

No electrical connections are required, and maintenance is only an annual change of prefilters, a carbon tower and a particulate final filter.

The physical size of the unit is 610 mm wide × 510 mm deep × 1750 mm high, and it weighs 114 kg.

*For more information, contact: Kelly Newvell, Whatman International Ltd., Whatman House, St Leonard's Road, 20/20 Maidstone, Kent ME16 0LS, UK; Tel: +44 (0)1622 6766670; Fax: +44 (0)1622 677011*

### New deuterium/tungsten light source for hand-held spectroscopy

A new, complete light source, Fiberlight, which has been purpose developed for hand-held, fibre-coupled UV-Vis spectrometers, is now available from Heraeus Noblelight Ltd, Cambridge. Fiberlight features a miniature deuterium lamp, for UV light, and a tungsten lamp, for visible light, with an integral shine-through arrangement and optical system.

Ultraviolet light from deuterium lamps is an essential component of any optical analysis technique, e.g. spectroscopy and high-pressure liquid chromatography, which measures the variation in the amount of UV or visible light absorbed by the sample under analysis. Deuterium lamps have always been used for these applications because of their high stability and the fact that they produce a line-free, continuous spectrum in the ultraviolet region. However, to date, size and power consumption have prevented these lamps being applied in hand-held analysis.

The miniature deuterium lamp of the new Fiberlight unit is an electrode-less high-frequency excited gas discharge lamp, of just 3 W power consumption, which generates negligible heat. The associated tungsten lamp also has a power requirement of ~ 3 W. The light exit from the unit is through an SMA fibre optic connector, and all elements of the unit, including shutter, optical system and 12 V DC power supply are mounted on a circuit board.

With its compact size, low weight and high-quality specification, Fiberlight is ideally suited to hand-held spectroscopy applications, e.g. water analysis, diagnosis and pollution control measurements. It can also be used with battery-driven spectrometers for field analysis and on-line process control.

Heraeus is one of the pioneers of UV lamp technology, especially of the deuterium lamp. It operates very closely with other researchers, industrial development engineers and instrumentation manufacturers to ensure continuous product development, not only in lamps but also in ancillaries and systems, e.g. the new Fiberlight light source. This total capability ensures that manufacturers and users of analytical instrumentation achieve optimum performance from highly sophisticated equipment.

Heraeus Noblelight Ltd, part of the Heraeus Group, specializes in the production and application of high-quality energy sources covering the entire electromagnetic spectrum, from ultraviolet to infra-red. It offers a range of UV lamps to match most of the UV/VIS Spectrometers and HPLC UV Detectors in use today.

*For more information contact Martin Woods, Heraeus Noblelight Ltd, Cambridge Science Park, Milton Road, Cambridge CB4 4GQ, UK. Tel: +44 (0) 1223 423324, Fax: +44 (0) 1223 423999*

### Meeting report

### *P S Analytical sets the standard at Pittcon 99*

At the Pittsburgh Conference in Orlando, Florida, P S Analytical introduced a number of exciting new products within its Millennium and Sir Galahad product ranges.

The Millennium Merlin/Galahad instrument has been introduced to fulfil the expectations of the EPA 631 methodology. The PSA 10.025 encorporates a gold amalgamation step into the already proven Millennium Merlin. The instrument can be used with either the standard PSA carousel autosampler or the new xyz model. The introduction of the amalgamation step increases the detection capabilities of the Merlin system with a sample throughput of ~10 samples per hour.

The standard Millennium Merlin has a detection range of <0.2 ppt quantity at a sample throughput of 30 samples per hour. One further unique features of this range is the ability to analyse samples in the 0–10 ppm range without any system modification.

Mercury, arsenic and selenium speciation are fast becoming significant, not only in the academic research area but also for future regulatory issues. The Millennium range of products can be readily integrated into chromatographic or purge-and-trap schemes to develop speciation profiles. To enable the products to do this, PSA has developed a simple retrofit 0–1 V option for the Millennium range, and is also able to provide the users with standard kits to enable speciation studies to be readily achieved.

In the petrochemical industry, there is also considerable interest in the determining presence of mercury either as mercury(0) or organic derivations. P S Analytical also introduced its PSA 10.515 preconcentration device to assist in the analysis of mercury levels in petrochemical condensates. This new instrument can handle 0–1 μl samples of condensates, and prepares samples prior to analysis by the PSA Sir Galahad instrument. Speciation profiles can be readily provided using the PSA 10.727 gas chromatography–atomic fluoresence system.

*Details of all of these new products are available from P S Analytical, Arthur House, Crayfields Industrial Estate, Main Road, Orpington, Kent BR5 3HP, UK. Tel: +44 (0)1689 891211; Fax: +44 (0)1689 896009; e-mail: sales@psanalytical.demon.co.uk; URL—http://www.psanalytical.com*

